# A first-in-human study of the novel metabolism-based anti-cancer agent SM-88 in subjects with advanced metastatic cancer

**DOI:** 10.1007/s10637-019-00758-8

**Published:** 2019-03-30

**Authors:** Jeanetta Stega, Marcus S. Noel, Alexander G. Vandell, Damian Stega, Giuseppe Del Priore, Steve Hoffman

**Affiliations:** 1 QRI LLC, 605 Majors Path, South Hampton, NY 11938 USA; 2 Division of Hematology/Oncology, James P. Wilmot Cancer Institute, University of Rochester Medical Center, 601 Elmwood Avenue, Box 704, Rochester, NY 14642 USA; 3 Tyme Technologies, Inc., 17 State St. 7th floor, New York, NY 10004 USA; 4 Morehouse School of Medicine, 720 Westview Drive, Atlanta, GA 30310 USA

**Keywords:** SM-88, Oncology, SMK therapy, Phase 1, Cancer metabolism

## Abstract

*Purpose* SM-88 (D,L-alpha-metyrosine; racemetyrosine) is a novel anti-cancer agent, used with melanin, phenytoin, and sirolimus (SMK Therapy). This pilot first-in-human study characterized the safety, tolerability, and efficacy of SMK Therapy in subjects with advanced metastatic cancer. *Methods* All subjects (*n* = 30) received SMK Therapy for an initial 6 week Cycle (5 days on, 2 off per week) and continued if well tolerated. Safety signals, clinical response, overall survival, progression free survival (PFS), and quality of life changes were assessed. *Results* The most common drug related adverse events were hyperpigmentation and rash. All drug related adverse events were mild to moderate in intensity. Following treatment with SMK Therapy, 4 subjects achieved complete response, 6 partial response, and 17 stable disease according to Response Evaluation Criteria in Solid Tumors (RECIST) 1.1 (total clinical benefit 90%). Responses were observed within 6 weeks, and continued to improve, with 3 complete and 3 partial responders achieving best response after at least 3.2 months. Durable stable disease was observed, lasting a median duration of 11 months (range 1–31 months). Median overall survival for all subjects was 29.8 months, and median PFS was 13 months. Following 6 weeks of treatment, most (83.3%) subjects showed an improvement in Eastern Cooperative Oncology Group (ECOG) score and an improvement in the European Organization for Research and Treatment of Cancer Quality of Life Questionnaire (EORTC QLQ 30) global health status (baseline 61.2 ± 25.0; end of Cycle 1 80.7 ± 14.7; *n* = 29; *p* < 0.001). *Conclusions* The results of this study support continued development of SM-88.

## Introduction

Metabolic reprogramming is a nearly ubiquitous aspect of tumor development that supports enhanced proliferation by increasing the supply of biochemical precursors, such as amino acids, lipids, and nucleotides [1]. Metabolic changes have also been shown to prevent activation of apoptosis [2] and to help tumor cells evade the immune response [3]. Important features of tumor metabolism include a preference for the conversion of glucose to ATP through glycolysis instead of oxidative phosphorylation even in the presence of an adequate oxygen supply (the Warburg effect) [4], and increased amino acid and lipid uptake for both energy metabolism and accumulation of biomass for replication [5–7]. There is increasing interest in both developing new and repurposing old drugs that target the altered metabolic state present in tumor cells. As metabolic changes in tumor cells are nearly universal (the Warburg effect has been reported in over 90% of tumors [8]), agents targeting these mechanisms have the possibility of exerting their therapeutic effect across a broad spectrum of different tumor types.

SM-88 (D,L-alpha-metyrosine; racemetyrosine) (Tyme Technologies, Inc., New York, NY) is a novel therapeutic agent designed to exploit the Warburg effect to selectively interfere with cancer cells’ protein synthesis pathways and increase oxidative stress. SM-88 was studied in use with three other drugs: melanin, phenytoin, and sirolimus (hereafter, referred to as SMK Therapy). The four components of SMK Therapy are hypothesized to work in a synergistic fashion to drive the targeted death of malignant cells. SM-88 is believed to directly interfere with cancer cells’ ability to synthesize critical proteins, including the protective transmembrane protein Mucin 1 (MUC1). The loss of MUC1 reduces the activity of intracellular anti-apoptotic pathways, increases sensitivity to reactive oxygen species (ROS) [9], and potentially exposes the cancer cell to immune recognition [10]. Sirolimus, though inhibition of mTOR is thought to increase insulin sparing cellular functions, thus forcing cancer cells to meet their metabolic demand by increasing uptake of amino acids and lipids [11, 12]. Phenytoin, through its induction of CYP3A4 can stimulate production of reactive lipid species [13–15], which may accumulate in the tumor microenvironment, increase the oxidative stress on the tumor, and help drive the cancer towards oxidative related apoptosis. Melanin, and other agents such as cisplatin and methoxsalen are recognized as electron donors, and in the presence of elevated tumor ROS concentrations, melanin may act as a catalyst promoting oxidative stress and facilitating free radical attack [16, 17].

We now report the first-in-human pilot study that was conducted to evaluate the safety and early efficacy signals of SMK Therapy in subjects with advanced metastatic cancers.

## Materials and methods

### Subjects

This study was conducted in accordance with the Declaration of Helsinki and in compliance with all International Conference on Harmonization Good Clinical Practice guidelines. The study protocol was approved by the New York Downtown Hospital Institutional Review Board on January 5, 2012. Informed consent was obtained from all individual participants included in the study.

Enrolled subjects met the following inclusion criteria: Aged ≥18 with confirmed progressive metastatic cancer who had either failed potentially curative treatment options or refused treatment with conventional chemotherapeutic agents; had an Eastern Cooperative Oncology Group (ECOG) performance status of 0 to 2 (higher ECOG scores were allowed at the discretion of the Sponsor and Investigator); had measurable disease by Response Evaluation Criteria in Solid Tumors version 1.1 (RECIST) [18]; had adequate renal function defined as having an estimated creatinine clearance ≥60 mL/min, serum creatinine <1.5 × upper limit of normal (ULN), and no known history of renal papillary necrosis or pyelonephritis; and had adequate hepatic function defined as bilirubin ≥1.5 × ULN, aspartate transaminase (AST) ≥2.5 × ULN (≥5.0 × ULN if hepatic metastases were present), and alanine transaminase (ALT) ≥2.5 × ULN (≥5.0 × ULN if hepatic metastases were present).

Subjects presenting with any of the following were excluded: Known leptomeningeal metastases, or symptomatic brain metastases. Subjects with previously treated brain metastases were eligible if treatment was completed and they had recovered from the acute effects of radiation therapy or surgery prior to the start of study regimen; received chemotherapy, radiotherapy (other than palliative radiotherapy to non-target lesions not being measured during this trial), biological, or investigational agents within 2 weeks of baseline disease assessments; underwent any surgery (not including minor procedures such as lymph node biopsy) within 4 weeks of baseline disease assessments, or not fully recovered from any side effects of previous procedures.

### Study design

This was a first-in-human, single-center, open-label, Phase 1 pilot study to evaluate the safety and efficacy of SMK Therapy in subjects with metastatic cancer.

The first subject was enrolled in January, 2012. Following completion of the first cycle, subjects who tolerated SMK Therapy were allowed to continue treatment for additional cycles, until disease progression or discontinuation for other reasons. Subjects were followed until September 19, 2017, when a final data analysis was performed. As of this data analysis, no subjects were receiving SMK Therapy.

The primary objective of this study was to evaluate the safety and tolerability of SMK Therapy. The secondary objectives of this study were to: Assess the progression-free survival (PFS) of subjects treated with SMK Therapy; assess measures of efficacy, including best overall response rate, duration of response, and overall survival; and to explore changes in health-related quality of life using patient-reported outcomes following treatment with SMK Therapy.

Oral SMK Therapy was administered as capsules consisting of 225 mg SM-88, 50 μg melanin, 15 mg phenytoin, and 0.2 mg sirolimus. Subcutaneous SMK Therapy was administered as two suspensions, one containing 5 mg SM-88, and the other containing 10 μg melanotan II, 2 mg phenytoin, and 0.05 mg sirolimus. Subcutaneous administration was included due to concern that subjects with late stage metastatic cancer may have impaired digestion, and thus unable to absorb an exclusively oral regimen. SMK Therapy was self-administered, under supervision, both orally and by subcutaneous injection each morning following an overnight fast on days 1–5 of each week for six weeks (one cycle). Subjects did not receive SM-88 on days 6 or 7 of each week.

Based on allometric scaling from pre-clinical data, and application of a safety factor of 1/6, the total starting dose of 230 mg (225 mg orally and 5 mg subcutaneously) SM-88 was chosen for this study. The doses for the other components of SMK Therapy were based on previous literature [19–23] and were expected to provide the previously described synergistic effects with SM-88 while minimizing the occurrence of adverse events.

Treatment was to be discontinued for progression of disease or intolerance (defined as Grade 4, Grade 3, or intolerable Grade 2 toxicity that did not return to Grade 1 or baseline after a 2-week interruption of treatment).

Dose limiting toxicity was defined any as Grade 4, Grade 3, or intolerable Grade 2 adverse event, except those clearly related to the disease, that did not return to Grade 1 or baseline after a 2 week interruption of treatment. At the discretion of the Investigator, up to 2 SM-88 dose reductions were allowed.

The number of subjects in this study was considered adequate to provide initial evidence of safety and to explore the clinical utility of SMK Therapy. This study was not powered for a efficacy endpoints.

### Assessments

Safety parameters included adverse events (AE), clinical laboratory evaluations, physical examination findings, and vital sign measurements. Adverse events were assigned Grades based on the National Cancer Institute (NCI) Common Terminology Criteria for Adverse Events (CTCAE) v 3.0.1. The reporting period for non-serious AEs terminated 28 days after the last dose of SMK Therapy or upon initiation of a subsequent anticancer treatment, whichever occurred first.

Tumor assessment, including physical examination and imaging scans (contrast-enhanced conventional or spiral computed tomography, or magnetic resonance imaging), was performed during Screening (or during a separate Baseline visit), at the end of Cycle 1 (6 weeks), and at 1 to 3 months post-Cycle 1 follow up visits. Subjects underwent an additional tumor assessment between Days 28 and 30 (end of week 4) of Cycle 1, if needed. For all subjects continuing to receive SMK Therapy beyond Cycle 1, additional follow up tumor assessments (including imaging) could be performed at the discretion of the Investigator throughout the duration of SMK Therapy.

Changes in quality of life were assessed using a battery of patient reported outcomes questionnaires, including the European Organization for Research and Treatment of Cancer Quality of Life Questionnaire (EORTC QLQ C30) and its Lung Cancer supplemental module (LC13) [24, 25], and the Dermatology Life Quality Index (DLIQ) [26]. Subjects completed the EORTC QLQ C30 and the LC13 module at screening and at the end of Cycle 1. Subjects completed the DLQI at screening, mid-Cycle 1, and at the end of Cycle 1. Subject’s DLQI scores were interpreted as follows: no effect on the subject’s life (score of 0–1), small effect (2–5), moderate effect (6–10), very large effect (11–20), and extremely large effect (21–30).

### Statistical analyses

The safety analysis set consisted of all subjects who received at least one dose of SMK Therapy, and was the primary analysis population unless otherwise mentioned. Survival status was obtained monthly during follow-up for all subjects. Survival status was tracked until the final data analysis, until the subject was deceased, declined continued participation, or was lost to follow up. All statistical analyses were performed in SAS version 9.3 (SAS Institute, Cary NC) or higher.

Best overall response was defined as the best response recorded (per RECIST 1.1) from the start of treatment until documentation of progressive disease and recorded as complete response (CR), partial response (PR) or stable disease (SD). Target lesions and tumor response were defined according to RECIST guidelines.

PFS was defined as the time from the first date of SMK Therapy administration to the date of disease progression, death due to any cause, or initiation of a new oncology treatment, whichever occurred first. Overall survival was defined as the interval from the first date of SMK Therapy administration to the date of death from any cause. Both PFS and overall survival were assessed using standard Kaplan-Meier methods. Subjects were censored at the time of last assessment for PFS. Subjects who were not confirmed to be deceased during follow up were censored at the last known time alive.

An exploratory analysis of penultimate PFS was performed based on previously described methods for subjects treated with platinum based therapies [27]. In brief, IRB approval was obtained and a full review of each subject’s medical history was conducted in order to obtain the date of initiation of the previous (penultimate) chemotherapy regimen and the subsequent date of first documented progressive disease. The duration of time between these two dates was considered to be the penultimate PFS for this analysis. PFS ratio was calculated by dividing the current PFS (SMK Therapy) by the penultimate PFS as described previously [28, 29].

## Results

### Demographics and baseline characteristics

A total of 30 subjects were enrolled and treated with SMK Therapy. Table 1 summarizes the demographics and baseline characteristics of all subjects enrolled in this study.

**Table 1:** Demographics and baseline characteristics

Characteristic	All Subjects (*N* = 30)
Sex, *n* (%)	
Female	21 (70.0%)
Male	9 (30.0%)
Age (Years)	
Mean ± SD	57.7 ± 9.87
Race, n (%)	
Asian	1 (3.33%)
Caucasian	29 (96.7%)
Weight (kg)	
Mean ± SD	68.4 ± 18.8
ECOG performance status, *n* (%)	
0	3 (10.0%)
1	14 (46.7%)
2	9 (30.5%)
3	3 (10.0%)
4	1 (3.33%)
Primary tumor type, *n* (%)	
Breast	14 (46.7%)
Lung	5 (16.7%)
Pancreas	3 (10.0%)
Prostate	2 (6.67%)
Colon	1 (3.33%)
Tongue	1 (3.33%)
Thyroid	1 (3.33%)
Liver (primary unknown)	1 (3.33%)
Appendix	1 (3.33%)
Biliary	1 (3.33%)
Prior radiotherapy, *n* (%)	11 (36.7%)
Prior surgery, *n* (%)	19 (63.3%)
Number of prior chemotherapy regimens, *n* (%)	
< 2	19 (63.3%)
≥ 2	11 (36.7%)

### Safety profile

In total, all 30 subjects experienced at least one treatment emergent adverse event (TEAE). The most common TEAEs experienced by ≥10% of subjects, are shown in Table 2. SMK Therapy related TEAEs occurring in ≥10% of subjects included hyperpigmentation (30 [100%]), fatigue (17 [56.7%]), and pain (3 [10.0%]). Fatigue was generally transient, usually resolving in 2–3 weeks. All SMK Therapy related TEAEs were classified as mild or moderate by the Investigator. Four severe adverse events (SAE) occurred during the study (decreased weight, edema, hip pain, generalized pain), each in 1 subject. No SAE was judged to be related to the SMK Therapy. No dose limiting toxicities were observed. No subject discontinued treatment, had a dose reduction, or experienced an interruption of SMK Therapy administration due to a TEAE. Three subjects (10.0%) discontinued therapy after completion of Cycle 1 due to clinical disease progression. As of the final data analysis, which included 38 months of follow up, 25 subjects (83.3%) had died. No deaths were deemed related to be SMK Therapy related.

**Table 2:** Summary of treatment emergent adverse events including those experienced by at least 10% of subjects (all cause), and proportion of SMK therapy related adverse events

TEAE	All Causes	SMK Therapy Related
All TEAEs	30 (100%)	30 (100%)
Hyperpigmentation	30 (100%)	30 (100%)
	29 (96.7%) Grade 1	29 (96.7%) Grade 1
	1 (3.3%) Grade 2	1 (3.3%) Grade 2
Fatigue	21 (70.0%)	17 (56.7%)
	17 (56.7%) Grade 1	13 (43.3%) Grade 1
	4 (13.3%) Grade 2	4 (13.3%) Grade 2
Pain	19 (63.3%)	3 (10.0%)
	13 (43.3%) Grade 1	2 (6.7%) Grade 1
	4 (13.3%) Grade 2	1 (3.3%) Grade 2
	2 (6.7%) Grade 3	
Nausea	9 (30.0%)	0 (0.0%)
	6 (20.0%) Grade 1	
	3 (10.0%) Grade 2	
Back Pain	5 (16.7%)	1 (3.3%)
	2 (6.7%) Grade 1	1 (3.3%) Grade 1
	3 (10.0%) Grade 2	
Diarrhea	4 (13.3%)	0 (0.0%)
	4 (13.3%) Grade 1	
Headache	4 (13.3%)	0 (0.0%)
	3 (10.0%) Grade 2	
	1 (3.3%) Grade 1	
Drowsiness	3 (10.0%)	0 (0.0%)
	3 (10.0%) Grade 1	

*n*(%) reported; Relationship of adverse event to the SMK Therapy was determined by the Investigator

### Assessment of therapeutic effect

**Fig. 1:**
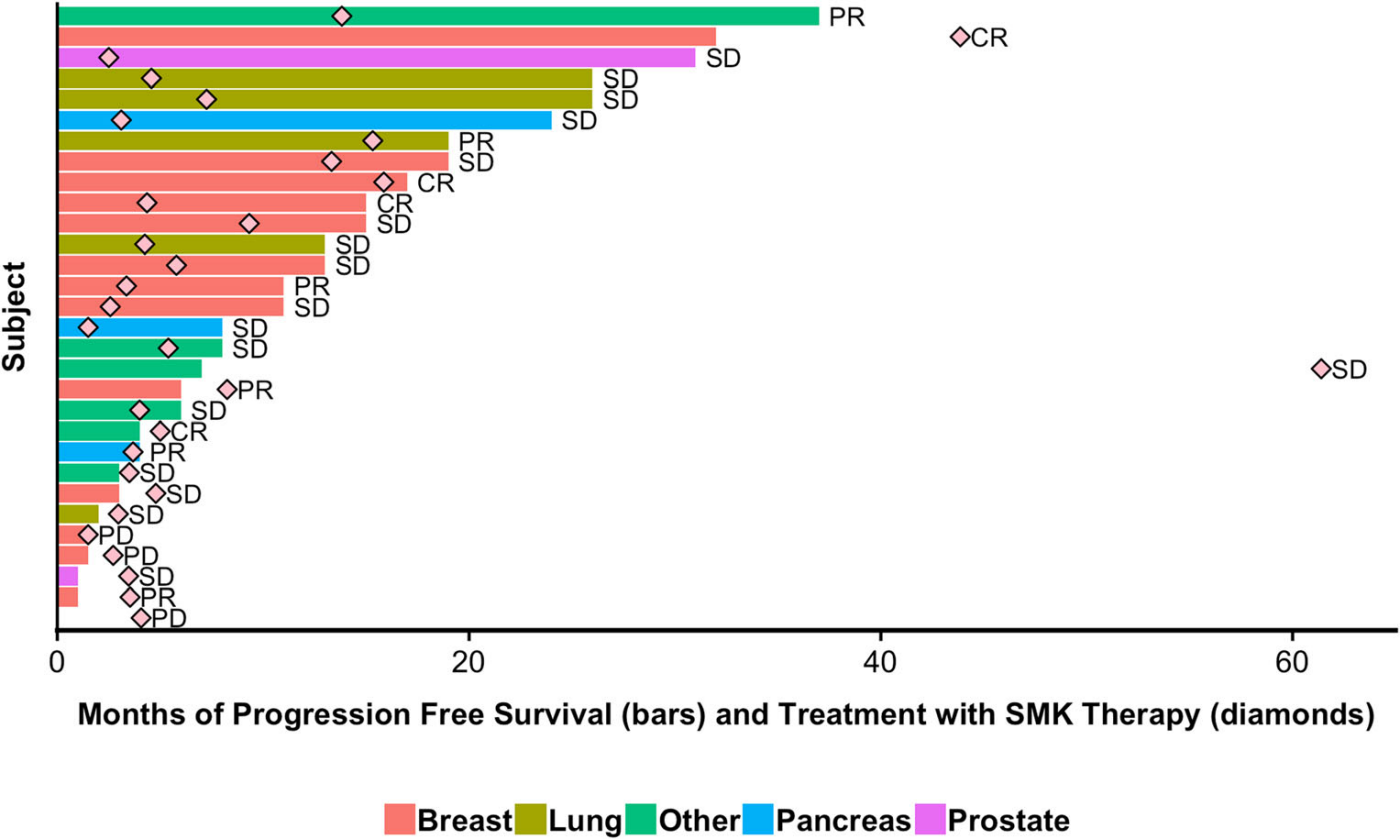
**Duration of treatment with SMK Therapy and PFS by subject.** The duration of time in months that each subject received SMK Therapy is shown by the diamond. Duration of PFS for each subject receiving SMK Therapy is shown by the bar, with the color of the bar indicating tumor type. The best response (progressive disease [PD], SD, PR, and CR) following SMK Therapy is indicated for each subject is also provided

Following treatment with SMK Therapy, clinical benefit (defined by RECIST 1.1 guidelines) was observed in 27 (90.0%) of subjects, including 4 subjects with CR, 6 with PR, and 17 with SD. Subjects remained on SMK Therapy for a median of 4.0 months (range: 1.4–57.3 months). Figure 1 displays the total duration of SMK Therapy and the PFS for each subject, by tumor type. Of note, two subjects continued to receive SMK Therapy for a year or more after disease progression. These subjects did not wish to pursue conventional chemotherapy or radiation therapy and requested that they continue to receive treatment with SMK Therapy, claiming that they felt better while on treatment. Per their request, they were allowed to remain on SMK Therapy as compassionate use.

Table 3 summarizes data for all 10 subjects that experienced either a PR or CR. Initial radiographic responses were often observed within the 6 weeks of treatment. Six of 10 subjects achieving PR or CR reached their best clinical response after at least 3.2 months of treatment with SMK Therapy. Interestingly, two of these subjects (a CR with cancer of the appendix, and a PR with thyroid cancer) experienced best overall response at least one month after discontinuing SMK Therapy, but before progressing to a new line of treatment.

**Table 3:** Summary of subjects with an overall response of PR or better

Subject (Age/Sex)	Tumor Type	Best Response	Time to Best Response (Months)	Duration of SMK Therapy (Months)	ECOG Score^*^ (Baseline / End of Cycle 1)	EORTC Global Health Status Score^#^ (Baseline / End of Cycle 1)
70/Female	Breast	CR	1.5	4.1	2/1	5/7
50/Female	Breast	CR	3.3	40.9	1/0	3/7
51/Female	Breast	CR	3.2	14.8	2/1	3/6
57/Male	Appendix	CR	5.6	4.7	1/0	5/7
40/Female	Breast	PR	1.4	7.7	3/1	4/6
54/Female	Breast	PR	1.4	3.3	2/1	7/6
58/Female	Lung	PR	4.1	14.3	2/1	4/5
58/Male	Pancreas	PR	3.4	3.4	2/1	4/5
48/Female	Breast	PR	1.5	3.1	1/0	4/6
33/Female	Thyroid	PR	15.5	12.9	1/0	3/7

*A reduction in ECOG score indicates improvement. #An increase in EORTC global health status score indicates an improvement

Four subjects experienced CRs following treatment with SMK Therapy. One CR occurred in a 57 year old male subject with appendiceal carcinoma, and had previously received 1 line of chemotherapy. Three CRs occurred in subjects with breast cancer. Of these, 2 subjects, 50 and 51 year old females, who received 2 and no previous lines of chemotherapy respectively, had breast cancers classified as ER(+)/PR(+)/HER2(−). The third, a 70 year old female, had breast cancer classified as ER(+)/PR(−)/HER2(−), and had previously received 4 lines of chemotherapy. All three individuals had bone metastases at baseline that appeared to completely resolve following treatment with SMK Therapy.

**Fig. 2:**
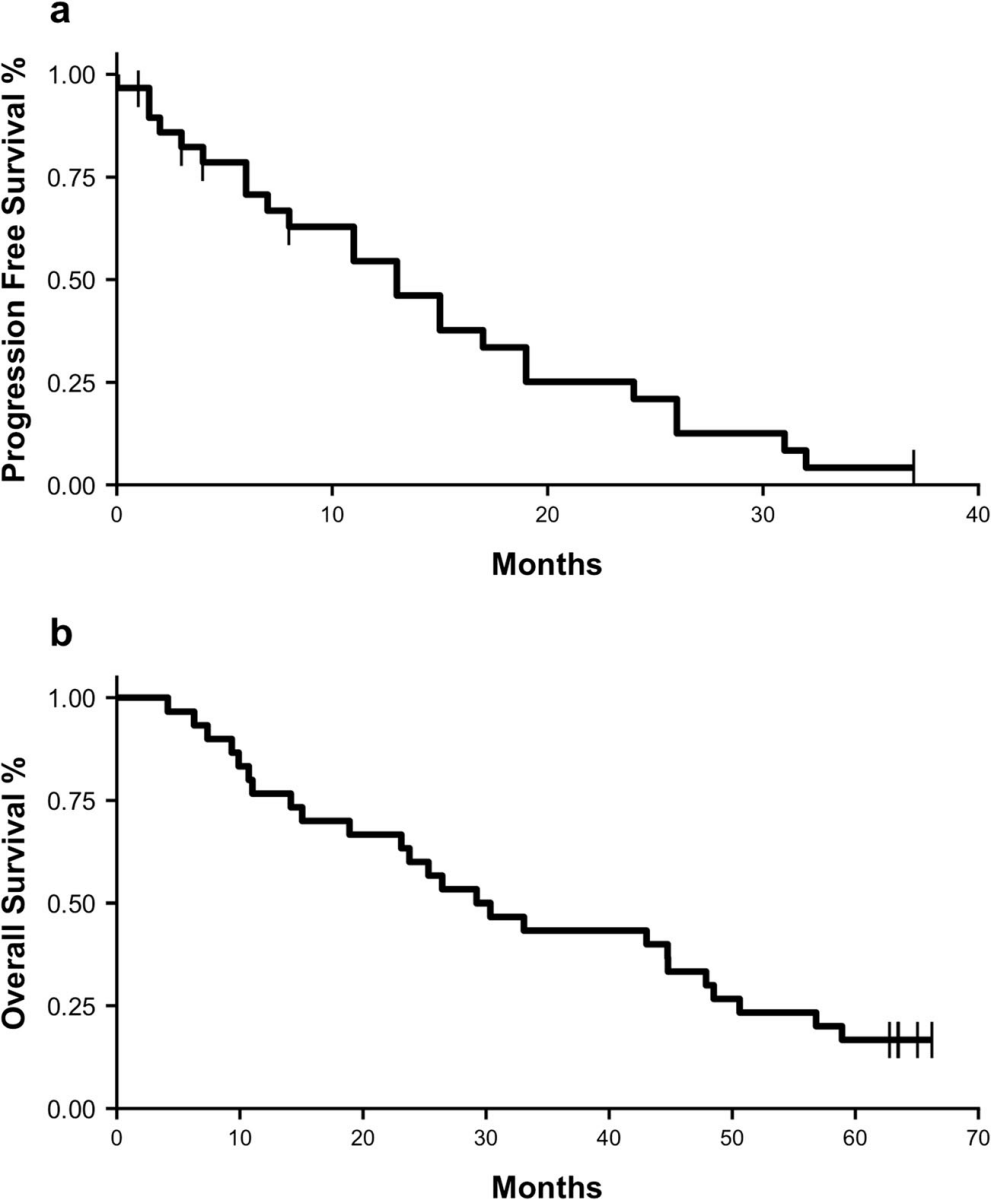
**Overall Survival and PFS following treatment with SMK Therapy.** Kaplan-Meier plots showing (**a**) overall survival, and (**b**) PFS for all subjects

PFS was determined for all subjects using Kaplan-Meier [KM] analysis and is displayed in Fig. 2a. Median PFS for all subjects was 13 months (95% CI: 8–24, KM estimate). Five (16.7%) subjects were censored at last radiographic follow up with no documented progression. An additional 9 subjects died without documented disease progression. Following treatment with SMK Therapy, 12 (40.0%) of subjects received subsequent chemotherapy or radiotherapy, 1 (3.3%) subject underwent surgical resection, and 1 (3.3%) subject continued with sporadic maintenance use of SMK Therapy.

Duration of response for all subjects was also determined by examining overall survival. Median overall survival for all subjects was determined by KM analysis and is shown in Fig. 2b. Median overall survival for all subjects was 29.8 months (95% CI: 23.1–48.5, KM estimate), with no subjects lost to follow up. Five (16.7%) subjects were still alive at the time of final data analysis.

Durable SD was observed in this study, with SD lasting for a median duration of 11 months (range 1–31 months) until disease progression, with a median duration of treatment of 4 months. Median overall survival of subjects achieving a best response of SD was 30.3 months (95% CI: 25.3–48.5, KM estimate).

**Fig. 3:**
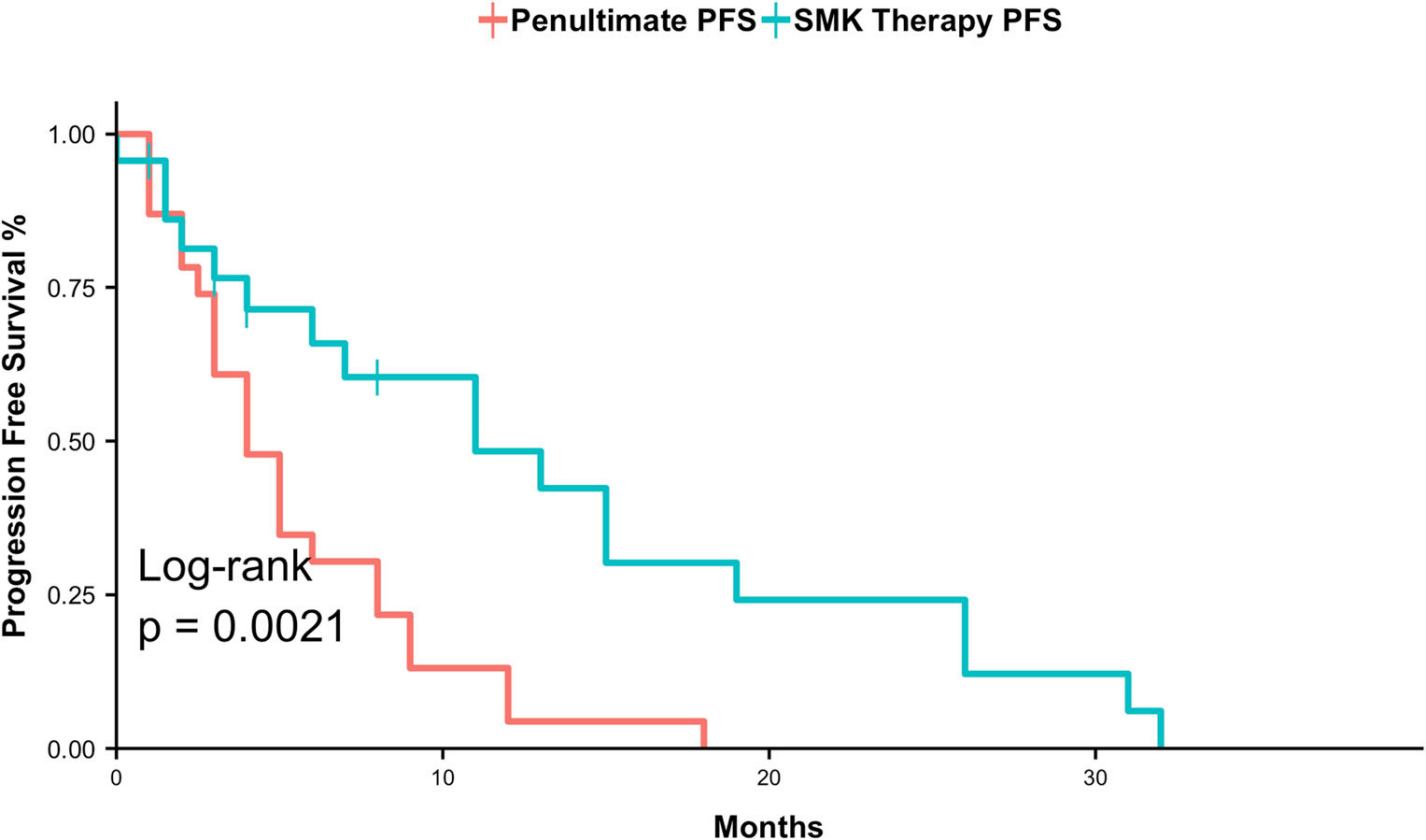
**PFS following treatment with SMK Therapy compared to penultimate PFS following prior chemotherapy regimens.** Kaplan-Meier plot comparing PFS following treatment with SMK Therapy (red) to penultimate PFS (blue). This analysis is based on the 23 subjects for which penultimate PFS data were available

**Fig. 4:**
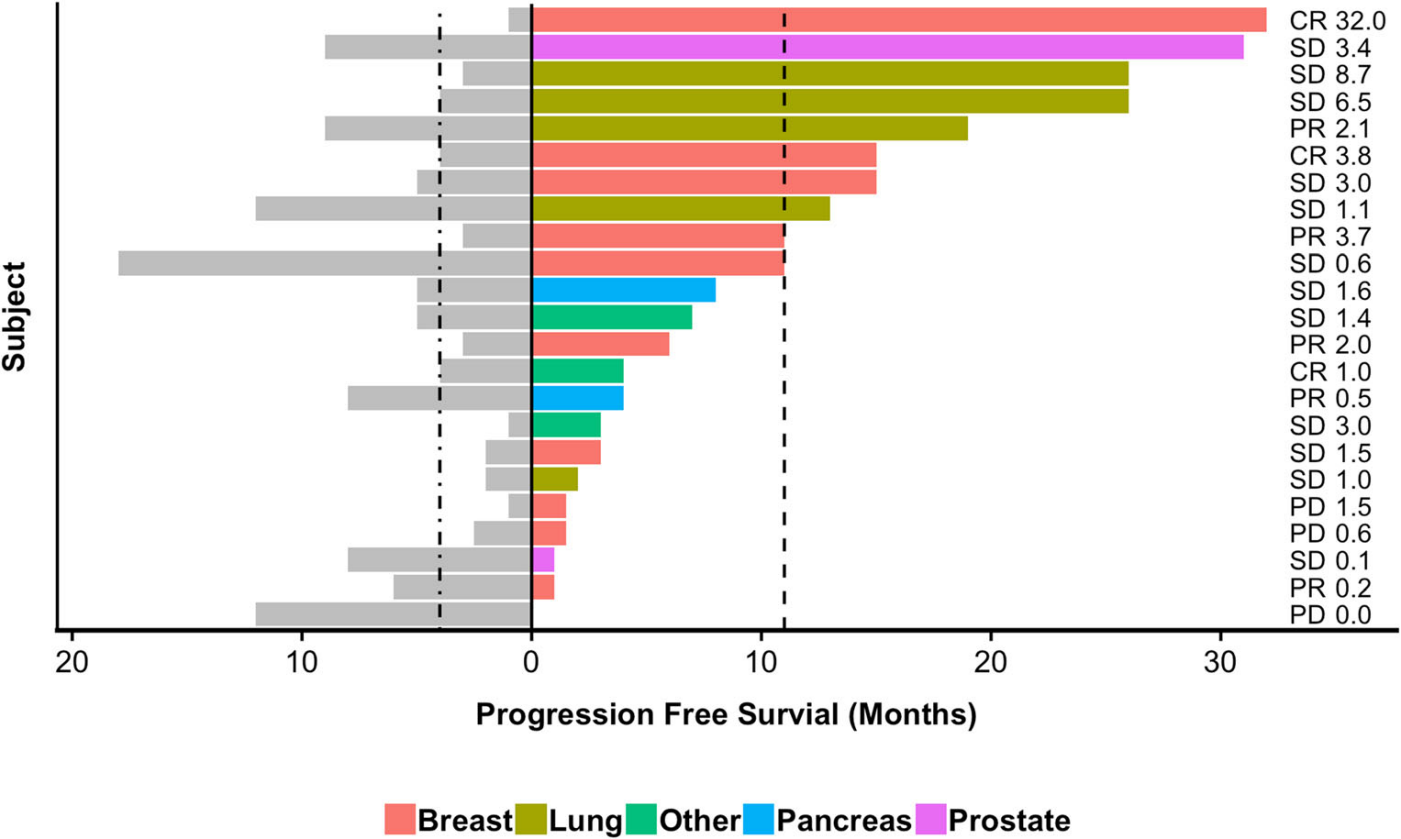
**PFS following treatment with SMK Therapy and penultimate PFS by subject.** For each of the 23 subjects for which penultimate PFS data were available, the duration of penultimate PFS is shown by the gray bars to the left. Colored bars to the right show PFS following treatment with SMK Therapy, with the colors indicating tumor type. Vertical dashed lines indicate the Kaplan-Meier estimated medians. The best response (progressive disease [PD], SD, PR, and CR) following SMK Therapy is indicated for each subject as is the penultimate PFS ratio (SMK Therapy PFS / penultimate PFS)

As there was no formal control group in this study, an exploratory analysis was conducted using each subject’s penultimate PFS for comparison. The last PFS period (penultimate PFS) before enrollment in a clinical trial represents the efficacy of standard of care treatment. Following a review of medical history, penultimate PFS data could be reliably determined for 23 subjects. Figure 3 displays a Kaplan-Meier plot comparing penultimate PFS to SMK Therapy PFS. The median penultimate PFS was 4 months (95% CI: 3–8, KM estimate) while the median SMK Therapy PFS was 11 months (95% CI: 6–26, KM estimate) (*p* = 0.0021, log-rank test). Figure 4 shows the penultimate PFS and PFS following treatment with SMK Therapy for each subject, the associated PFS ratio (SMK Therapy PFS / penultimate PFS), and the best overall response to SMK Therapy. The median PFS ratio was 1.45, with 13 of 23 (56.5%) subjects having PFS ratios above 1.42, which suggests that SMK Therapy may provide clinical benefit [28, 29].

### ECOG response assessment

Table 4 summarizes the distribution of ECOG scores collected at each assessment (baseline, at mid-Cycle 1, and at the end of Cycle 1). A majority of subjects (*n* = 19, 63.3%) showed an improvement in ECOG score by mid-Cycle 1, and most subjects (*n* = 25, 83.3%) showed improvement by the end of Cycle 1. There were no deteriorations of ECOG performance status during the trial.

**Table 4:** Summary of change in ECOG scores following treatment with SMK therapy

ECOG Score	Screening N (%)	Mid-Cycle 1 N (%)	End of Cycle 1 N (%)
0	3 (10.0%)	12 (40.0%)	15 (50.0%)
1	14 (46.7%)	9 (30.0%)	14 (46.7%)
2	9 (30.0%)	8 (26.7%)	1 (3.33%)
3	3 (10.0%)	1 (3.33%)	0 (0.00%)
4	1 (3.33%)	0 (0.00%)	0 (0.00%)
5	0 (0.00%)	0 (0.00%)	0 (0.00%)
Mean Score	1.5	0.93	0.53

### Quality of life assessments

The primary instrument employed to assess changes in quality of life based on patient reported outcomes was the EORTC QLQ 30. Table 5 summarizes the baseline and end of Cycle 1 scores for each item on the EORTC QLC 30. There was a statistically significant improvement in the EORTC QLQ 30 global health status item following 6 weeks of treatment with SMK Therapy (baseline 61.2 ± 25.0; end of Cycle 1 80.7 ± 14.7; *n* = 29; *p* < 0.001). No meaningful changes were observed for any item in the LC13 module following six weeks of treatment with SMK Therapy (data not shown).

**Table 5:** Summary of change in EORTC QLC 30 scores following treatment with SMK therapy, by item

Parameter	Screening	End of Cycle 1
Global Health Status		
Global health status	61.2 ± 25.0	80.7 ± 14.1
Functional Scales		
Physical functioning	77.1 ± 21.5	87.6 ± 14.2
Role functioning	74.1 ± 29.1	85.6 ± 23.8
Emotional functioning	76.1 ± 23.0	85.9 ± 17.1
Cognitive functioning	76.4 ± 25.4	85.1 ± 22.4
Social functioning	72.4 ± 32.5	83.3 ± 24.0
Symptom Scales		
Fatigue	39.1 ± 26.8	26.8 ± 24.4
Nausea and vomiting	10.9 ± 21.9	5.75 ± 12.8
Pain	35.1 ± 31.3	17.2 ± 22.9
Dyspnea	18.4 ± 24.5	10.3 ± 10.3
Insomnia	25.3 ± 32.9	11.5 ± 20.5
Appetite loss	19.5 ± 30.2	10.3 ± 22.0
Constipation	12.6 ± 24.3	9.20 ± 21.6
Diarrhea	10.3 ± 18.0	9.20 ± 17.6
Financial difficulties	31.0 ± 36.7	25.3 ± 30.4

*N* = 29; Mean and SD are provided

The DLQI was used to assess changes in quality of life related to changes in skin tone resulting from the melanin component of SMK Therapy. Table 6 summarizes the reported quality of life at each DLQI assessment. Overall, SMK Therapy did not appear to affect quality of life as measured by the DLQI.

**Table 6:** Summary of change in DLIQ scores following treatment with SMK therapy

Effect on Quality of Life	Screening *N* (%)	Mid-Cycle 1 *N* (%)	End of Cycle 1 *N* (%)
None	28 (93.3%)	27 (90.0%)	26 (86.6%)
Small	0 (0.00%)	2 (6.67%)	3 (10.0%)
Moderate	2 (6.67%)	1 (3.33%)	1 (3.33%)
Very Large	0 (0.00%)	0 (0.00%)	0 (0.00%)
Extremely Large	0 (0.00%)	0 (0.00%)	0 (0.00%)

Classifications of the effect on quality of life were determined based on DLQI score

## Discussion

This pilot first-in-human study examined the safety and efficacy of SM-88, a novel anti-cancer agent used with melanin, phenytoin, and sirolimus, in subjects with advanced cancers. Overall, SMK Therapy was safe and well tolerated with no major safety signals identified. All drug related adverse events were mild to moderate (Grade 1 or 2) in severity, with hyperpigmentation and fatigue predominating. Hyperpigmentation was an expected complication secondary to administration of melanin and melanotan II. While hyperpigmentation occurred in all subjects, based on the DLIQ it did not affect subjects’ quality of life. Fatigue was also expected as it is a well-known side effect of the L-alpha-metyrosine isomer, which is used in the treatment of pheochromocytoma [30], and was generally observed to be transient.

SMK Therapy demonstrated possible efficacy across a variety of tumor types, with 4 CRs, 6 PRs, and 17 SDs observed. The total clinical benefit was notable, with 90% of subjects achieving a best overall response of SD or better, and most subjects reporting improvements within the first 6 weeks of treatment. Based on typical breast cancer genomic markers (ER, PR, and HER2) and preliminary data from the ongoing Phase 1b/2 study in prostate cancer [31], the mechanism of action of SMK Therapy does not appear to be dependent on hormonal or HER2 status. This is further supported by the PR observed in one subject with triple-negative breast cancer.

In a majority of subjects achieving a PR or CR, best clinical response was observed only after at least 3.2 months of treatment with SMK Therapy, suggesting that long-term administration may be need for SM-88 to achieve its full therapeutic potential. SMK Therapy may also exhibit a carry-over response as suggested by the two subjects whose best clinical responses were documented at 1 and 3 months, respectively, after cessation of SMK Therapy but before a new treatment was initiated. Of note, durable clinical benefits were not limited to only those subjects achieving CR or PR, but were also present in those subjects achieving SD. Durability of response is further supported by PFS data, with 7 subjects exhibiting responses lasting ≥12 months, and an additional 6 subjects with responses lasting ≥24 months.

Both the occurrence of delayed responses and durable clinical benefit were not unanticipated, and may be related to the proposed mechanism of action of SMK Therapy. SMK Therapy is not thought to be immediately cytotoxic, instead interfering with protein synthesis and other cellular mechanisms to increase the burden of ROS in the cancer cell, akin to radiation therapy. Interestingly, oxidative stress induced by radiation therapy was a stated underpinning of the mechanism of action in designing SMK Therapy. Additional clinical, animal, and in vitro studies will be needed to confirm these results and further define the underlying mechanisms.

At least 2 responders (1 CR and 1 PR) had documented local radiotherapy within 3 months of initiating treatment with SMK Therapy. As the response to radiotherapy may occur over the course of several weeks or months, it is possible that these responses are not due solely to the activity of SMK Therapy. They could be driven by radiotherapy, or by synergistic activity between radiotherapy and SMK Therapy. However, even if these cases were excluded from the analysis, the direction of the results would not have changed.

Results from the exploratory penultimate PFS analysis suggest that SMK Therapy may be at least as efficacious as standard of care, with the median PFS following treatment with SMK Therapy being almost triple that of the penultimate PFS observed following standard of care. The potential efficacy of SMK Therapy is further supported by the PFS ratio, an emerging metric which has been associated with clinical benefit [28, 29]. Although the results of this analysis are encouraging, they should be interpreted cautiously. First, standard of care for many cancers has improved in the more than 5 years since this study began. Second, penultimate PFS is determined through examination of past medical records which may be incomplete or inaccurate. Finally, the analysis was not sufficiently powered, and thus should be considered preliminary.

Improvement in patient reported outcomes parallels those observed radiographically. Both the ECOG score and EORTC QLQ 30 global health status item improved over the first 6 weeks, further supporting the possible clinical efficacy of SMK Therapy. Given the duration of time that subjects remained on SMK Therapy, the median PFS and overall survival observed following treatment with SMK Therapy, and potential carry-over effect following treatment with SMK Therapy, it is likely that subjects experienced a long-term subjective improvement in quality of life. Unfortunately, the questionnaires were not administered in later cycles so we cannot accurately gauge the magnitude and extent of this improvement.

As a pilot study, there were significant operational limitations on the number and extent of analyses that were able to be conducted. In particular, pharmacokinetic and pharmacodynamic sample collection and analysis were not performed, and thus it is not possible to comment on the extent of exposure to SMK Therapy, or on any potential exposure-response relationship.

Results from this first-in-human study suggest that proof-of-concept with SMK Therapy has been achieved. Based on the safety profile and clinical efficacy of SMK Therapy observed in this first-in-human study, two clinical trials of SM-88 were initiated and are presently ongoing, a Phase 1b/2 study in subjects with prostate cancer (NCT02796898) and a Phase 2 study in subjects with pancreatic cancer (NCT03512756). In these studies, SM-88 is used with methoxsalen, phenytoin, and sirolimus (referred to as SM-88 Therapy), with methoxsalen playing a similar role to that of melanin and melanotan II in SMK Therapy. SM-88 Therapy also offers an additional advantage over SMK Therapy by eliminating the use of subcutaneous injections. Preliminary results from these trials are encouraging and have been reported [31].
